# Electrodeposited rGO/AuNP/MnO_2_ Nanocomposite-Modified Screen-Printed Carbon Electrode for Sensitive Electrochemical Sensing of Arsenic(III) in Water

**DOI:** 10.3390/bios13050563

**Published:** 2023-05-21

**Authors:** Yanqing Wu, Tao Zhang, Lishen Su, Xiaoping Wu

**Affiliations:** Key Laboratory for Analytical Science of Food Safety and Biology (Ministry of Education & Fujian Province), College of Chemistry, Fuzhou University, Fuzhou 350116, China; lalalawuuu@163.com (Y.W.); taozhang202304@163.com (T.Z.); 201310017@fzu.edu.cn (L.S.)

**Keywords:** electrochemical sensor, gold nanoparticles, metal oxides, nanocomposites, reduced graphene oxide, screen printed carbon electrode, arsenic

## Abstract

Herein, a simple and portable electrochemical sensor based on a reduced graphene oxide/gold nanoparticle/manganese dioxide (rGO/AuNP/MnO_2_) nanocomposite-modified screen-printed carbon electrode (SPCE) was constructed by the facile stepwise electrodeposition method and used for electrochemical detection of As(III). The resultant electrode was characterized for its morphological, structural, and electrochemical properties using scanning electron microscopy (SEM), X-ray photoelectron spectroscopy (XPS), energy dispersive X-ray spectroscopy (EDX), cyclic voltammetry (CV), and electrochemical impedance spectroscopy (EIS). From the morphologic structure, it can be clearly observed that the AuNPs and MnO_2_ alone or their hybrid were densely deposited or entrapped in thin rGO sheets on the porous carbon surface, which may favor the electro-adsorption of As(III) on the modified SPCE. It is interesting that the nanohybrid modification endows the electrode with a significant decrease in charge transfer resistance and an increase in electroactive specific surface area, which dramatically increases the electro-oxidation current of As(III). This improved sensing ability was ascribed to the synergistic effect of gold nanoparticles with excellent electrocatalytic property and reduced graphene oxide with good electrical conductivity, as well as the involvement of manganese dioxide with a strong adsorption property in the electrochemical reduction of As(III). Under optimized conditions, the sensor can detect As(III) via square wave anodic stripping voltammetry (SWASV) with a low limit of detection of 2.4 μg L^−1^ and a linear range of 25–200 μg L^−1^. The proposed portable sensor shows the advantages of a simple preparation procedure, low cost, good repeatability, and long-term stability. The feasibility of rGO/AuNPs/MnO_2_/SPCE for detecting As(III) in real water was further verified.

## 1. Introduction

Arsenic (As) is a highly toxic metalloid and is widely distributed throughout the environment in water and soil. Due to the widespread use of arsenic-containing compounds in agricultural and industrial processes, elevated levels of inorganic arsenic including As(III) and As(V) are found in contaminated groundwater and drinking water [1]. Especially, as the most stable and toxic form, As(III), has a stronger capacity for cellular uptake and accumulation than As(V) in the process of arsenic poisoning. In particular, its trivalent metabolites have a high affinity for sulfhydryl groups of proteins during the methylation detoxification of arsenic, leading to severe damage to cells and tissues [2]. Long-term ingestion of arsenic, mainly through contaminated drinking water or food, will lead to serious skin diseases, organ, and nervous system damage, and even carcinogenesis, thus posing a great threat to public health. The International Agency for Research on Cancer (IARC) has officially classified arsenic as a first-class carcinogen to humans. The current guideline level of arsenic in drinking water was set at 10 μg L^−1^ by the World Health Organization (WHO). In addition, groundwater arsenic contamination is usually higher than this limit in many countries, resulting in practical difficulties in removing arsenic from drinking water. It is urgent to develop a simple, sensitive, and rapid method to detect and monitor trace arsenic in water.

Arsenic detection using traditional analytical techniques, such as atomic emission spectrometry (AES) [3], atomic fluorescence spectrometry (AFS) [4], inductively coupled plasma–mass spectrometry (ICP-MS) [5], and atomic absorption spectrometry (AAS) [6], have been widely used and have shown high accuracy and sensitivity. However, they generally require bulky and expensive equipment as well as time-consuming sample pretreatment, which is not suitable for the rapid in situ detection of arsenic. Moreover, they are indiscriminate to the different charge states of inorganic arsenic. By contrast, electrochemical techniques, particularly anodic stripping voltammetry (ASV), provide a potentially sensitive, portable, simply-operated, and low-cost alternative for onsite rapid detection [7]. In addition, ASV has been identified as the most valuable technique in the trace analysis of arsenite since it can distinguish the different arsenic oxidation states via the sequential preconcentration and stripping of As(0) on the electrode surface [8].

Owing to their unique electric and catalytic properties, high surface area, and tunable surface chemistry, a number of nanomaterials have been utilized for electrode surface modification to improve the sensitivity and selectivity of As(III) detection, such as carbon nanostructures [9,10], metal oxides [11,12], noble metal nanoparticles [13,14], and bimetallic materials. Au-based nanomaterials have been recognized as an ideal substrate in the electrochemical detection of As(III) due to the formation of a strong As–Au intermetallic alloy [15,16]. However, the high cost and the possible agglomeration of gold nanoparticles hold up their reproducibility and field deployability [17]. The incorporation of Au-based nanocomposite materials on the electrode surface has become a feasible choice to resolve the inherent problems and obtain efficient functionality. In recent decades, several types of nanomaterials, including carbon nanostructures (carbon nanotubes [18], graphene [10]), metal oxides (iridium oxide nanotube [19], magnetic Fe_3_O_4_ nanoparticles [20], tin dioxide nanosheets [21], and cobalt oxide nanoparticles [22]), and conductive polymers (polyaniline [23], polypyrrole [24]) have attracted increasing attention in the development of nanocomposite sensors for arsenic, owing to their excellent high specific surface area, individual electrical/magnetic properties, catalytic properties, and cost-effectiveness. As a single atomic layer material with ultra-thin thickness, reduced graphene oxide (rGO) has been used to promote the dispersion of gold nanoparticles on the electrode surface along with the conductivity, which significantly improves the detection sensitivity of arsenic [25,26,27]. However, these rGO/AuNP-modified electrodes still suffer from the long deposition time for the As(III) pre-enrichment and the short service life of the sensor, which are associated with the lower adsorption capability and stability of the modifiers and may lead to a lack of practical applicability [16,25,28]. Recently, manganese oxide nanomaterials have attracted extensive attention in the field of electrochemistry and contamination removal because of their abundance, low price, and high adsorption capability and stability. To overcome the limitation in terms of electrical conductivity, the recombination of manganese oxides adsorbent with other materials such as noble metals or graphene is practicable for enhancing the sensing performance to As(III) [29,30]. It is well known that the sensing performance of an electrode largely depends on its electrocatalytic property and adsorption ability, the effective integration of complementary properties of different nanomaterials on the electrode interface will be beneficial to enhance its sensitivity, selectivity, and stability. It is expected that the effective combination of manganese oxides into the graphene modifier will improve the sensing efficiency and stability of Au-based nanocomposite-modified electrodes for As(III).

Compared to conventional electrodes, screen printing carbon electrode (SPCE) has the advantages of being small in size, low-cost, and disposable, and can easily be used in combination with portable heavy metal analysis equipment for on-site monitoring. By doping of graphitic defects, the selectivity of SPCE can be improved since the electrode substrate becomes less interactive and the electrocatalytic response toward analytes is ignorable [31]. Thereby, the fabrication of a sensitive and stable surface modification layer on the SPCE is highly required for the wide application of a rapid detection method for As(III).

In view of the need for a sensitive, stable, and low-cost sensor for arsenite monitoring in waters, herein we reported an in situ electrodeposition strategy of rGO/AuNP/MnO_2_ nanocomposites on the surface of SPCE for As(III) detection by square wave anodic stripping voltammetry (SWASV). The morphology and electrochemical behavior of the fabricated electrode were investigated. The proposed sensor was thoroughly examined to optimize the preparation and voltametric analytical parameters, as well as to evaluate the interference and reproducibility. In addition, the applicability of the sensor in the detection of As(III) in water samples was studied.

## 2. Experimental

### 2.1. Reagents and Materials

Chloroauric acid (HAuCl_4_, 99.9%) was purchased from HWRK Chemical Co., Ltd. (Beijing, China). Graphene oxide (GO, Lamellar classic) was purchased from Nanjing Xianfeng Nanotech Co., Ltd. (Nanjing, China). Manganese acetate tetrahydrate (C_4_H_6_MnO_4_•4H_2_O) was purchased from Sinopharm Chemical Reagent Co., Ltd. (Shanghai, China). As(III) standard solution (1000 mg L^−1^) was obtained from Aladdin Industrial Co., Ltd. (Shanghai, China). All other reagents obtained from Sinopharm Chemical Reagent Co., Ltd. (Shanghai, China), including sodium carbonate (Na_2_CO_3_), sodium bicarbonate (NaHCO_3_), 0.1 mol L^−1^ Na_2_CO_3_/NaHCO_3_ buffer solution (CBS, pH 9.0), sulfuric acid (98%), diethylene triamine pentaacetic acid (DTPA), ethylenediaminetetraacetic acid (EDTA), potassium ferricyanide (K_3_Fe(CN)_6_), sodium sulfate anhydrous (Na_2_SO_4_), potassium chloride (KCl), hydrochloric acid (HCl), and ethanol, were of analytical grade and used without further treatment. A 0.01M H_2_SO_4_ solution was used as the supporting electrolyte for SWASV analysis. All working solutions were prepared with ultrapure water (18 MΩ•cm) purified from a Milli-Q water purification system (Millipore, Burlington, MA, USA). Screen printing carbon electrodes (Φ = 3 mm, 0.071 cm^2^) were supplied by the Zensor R&D Co., Ltd. (Taiwan, China), consisting of carbon as the working electrode, carbon as the counter electrode, and silver as the reference electrode. All experiments were performed at controlled room temperature (25 ± 2 °C).

### 2.2. Apparatus

Electrochemical characterizations were carried out at a CHI 660 A electrochemical workstation (Chenhua, Shanghai, China). The surface morphology of the working electrodes was investigated using field-emission scanning electron microscopy (FE-SEM, Nova Nano SEM 230, Thermo Fisher Scientific, Waltham, MA, USA), high-resolution field-emission scanning electron microscopy (HRFE-SEM, Verios G4, Thermo Fisher Scientific, Waltham, MA, USA), and X-ray Photoelectron Spectroscopy (XPS, Escalab 250, Thermo Fisher Scientific, Waltham, MA, USA), and the elemental composition of the electrode surface materials was analyzed by energy dispersive X-ray spectroscopy (EDX, Quanta 250, Thermo Fisher Scientific, Waltham, MA, USA).

### 2.3. Fabrication of the Nanocomposite-Modified Electrode

**Figure 1:**
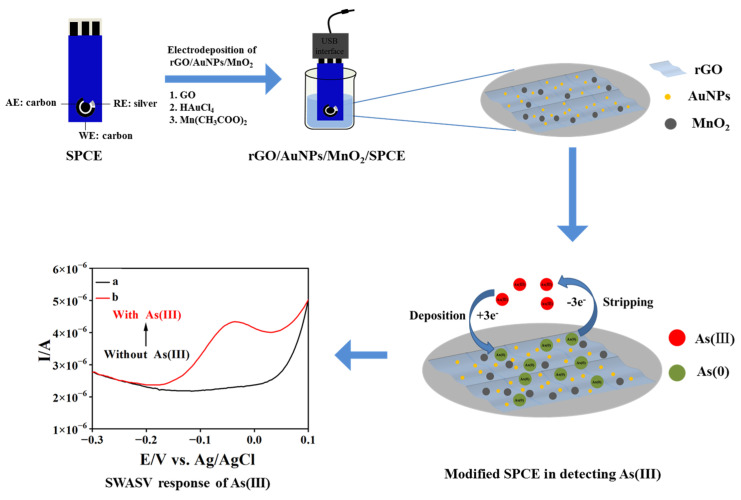
Schematic representation of the construction process of the rGO/AuNPs/MnO_2_-modified electrode and its sensing of As(III) by SWASV.

As illustrated in Figure 1, the rGO/AuNP/MnO_2_ nanocomposite-modified SPCE was fabricated via a step-by-step electrochemical deposition. Firstly, rGO was electrodeposited onto SPCE in 1.0 mg mL^−1^ graphene oxide dispersion (0.1 mol L^−1^ CBS (pH 9.0)) for eight cycles at a scan rate of 100 mV s^−1^ in the potential window of −1.5 V to 0.5 V. Then, the rGO/SPCE was immersed in 1.0 mmol L^−1^ HAuCl_4_ (0.2 mol L^−1^ Na_2_SO_4_) solution and deposited at a constant potential of −0.2 V for 90 s to obtain rGO/AuNPs/SPCE. Further, MnO_2_ was deposited onto rGO/AuNPs/SPCE in 0.05 mol L^−1^ Mn(CH_3_COO)_2_ (0.1 mol L^−1^ Na_2_SO_4_) at different deposition potentials (from −1.5 V to 0 V) and a scan rate of 50 mV s^−1^ for two cycles. Finally, the as-prepared rGO/AuNP/MnO_2_-modified SPCE was cleaned with ultrapure water and dried in N_2_ flow. For comparison, rGO/SPCE and rGO/AuNPs/SPCE were prepared, respectively, according to the above-mentioned electrode modification procedure.

### 2.4. Electrochemical Measurements

The electrochemical performance of rGO/AuNPs/MnO_2_/SPCE was studied by cyclic voltammetry (CV) and electrochemical impedance spectroscopy (EIS). The experimental parameters for CV were set as follows: −0.4 to 0.6 V, 100 mV/s. The experimental parameters for EIS were set as follows: 0.1~100 kHz. A 5 mmol L^−1^ [Fe (CN)_6_]^3−/4−^ solution containing 0.1 mol L^−1^ KCl was used for electrochemical characterization.

The electrochemical sensing behavior of As(III) was studied with SWASV under optimized conditions. In SWASV, As(III) species were initially reduced to As(0) on the electrode surface at −1.4 V for 180 s. The anodic stripping of electrodeposited As(0) was performed in 0.01 mol L^−1^ H_2_SO_4_ by a potential scan from −0.7 to 0.2 V at a potential step of 4 mV, an amplitude of 25 mV, and a frequency of 25 Hz. After each stripping step, the rGO/AuNPs/MnO_2_/SPCE was renewed in 0.01 mol L^−1^ H_2_SO_4_ by applying a preconditioning potential of 0 V for 15 s to remove adsorbed As(0). In addition, three replicate experiments were carried out for each SWASV determination, and all data were collected and used to estimate the error, taking into account the differences between the experiment.

### 2.5. Detection of Water Samples

To verify the practicability of proposed sensor, various drinking water samples, including tap water, mountain spring water, and mineral water, were obtained from Fuzhou University and Wanjia Supermarket (Fuzhou University, Fuzhou, China), respectively, and their basic chemical composition is shown in Table S1. The water samples were filtered through a 0.22 μm filter membrane. These samples were mixed with 0.01 mol L^−1^ H_2_SO_4_ in a volume ratio of 10:1 and determined by SWASV, respectively. Water samples spiked by three concentration levels of As(III) (50.0, 100.0, 150.0 μg L^−1^) were used for recovery experiments.

## 3. Results and Discussion

### 3.1. Preparation and Characterization of Nanocomposite-Modified Electrodes

Figure S1A–C show the cyclic voltammograms associated with the preparation of rGO/SPCE, rGO/AuNPs/SPCE, and rGO/AuNPs/MnO_2_/SPCE by stepwise electrodeposition, respectively. For rGO/SPCE, when the potential scanned from 0.5 to −1.5 V, a large reduction peak was observed at −1.1 V with a starting potential of −0.6 V, which can be ascribed to the reduction of oxygen-containing functional groups in GO (-OH, -COOH, and epoxides) [32]. In the reverse scanning, a small oxidation peak appears at −0.8 V, referring to the re-oxidation of reduced graphene. When the rGO/SPCE was potentiostated at −0.2 V in 0.2 mol L^−1^ Na_2_SO_4_ containing 1.0 mmol L^−1^ HAuCl_4_, the deposition of AuNPs on the electrode surface occurred and was completed at 90 s. As shown in Figure S1C, an enhancement of MnO_2_ deposition on the rGO/AuNPs/SPCE through a series of reactions was observed. In this case, an oxidation peak at −0.2 V can be attributed to the electrochemical oxidative deposition of Mn^2+^ to MnO_2_ [33,34], while the reduction peaks at −0.5 V and −1.3 V correspond to the re-reduction of MnO_2_ to Mn(OH)_2_ and the further conversion of Mn(OH)_2_ to Mn^2+^ (Equations (1)–(4)).

2H_2_O + 2e^−^ = H_2_ + 2OH^−^ (1)

O_2_ + 2H_2_O + 4e^−^ = 4OH^−^ (2)

Mn^2+^ + 2OH^−^ = Mn(OH)_2_ (3)

2Mn(OH)_2_ + O_2_ = MnO_2_ + H_2_O (4)

**Figure 2:**
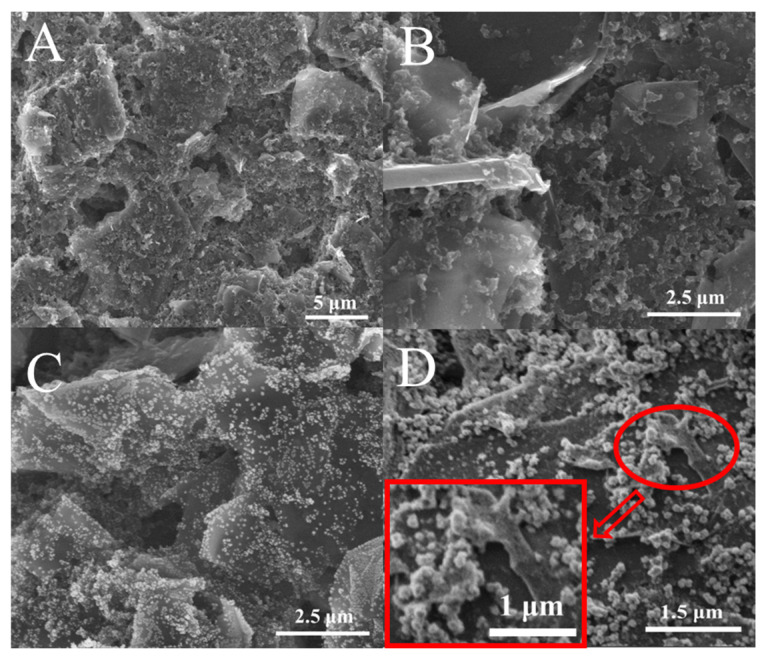
SEM images of bare SPCE (**A**), rGO/SPCE (**B**), rGO/AuNPs/SPCE (**C**), and rGO/AuNPs/MnO_2_/SPCE with an enlarged partial view (**D**).

The morphologies of bare SPCE, rGO/SPCE, rGO/AuNPs/SPCE, and rGO/AuNPs/MnO_2_/SPCE were investigated using SEM. As shown in Figure 2A, a porous structure with the flake-like morphology of carbon was observed on the bare SPCE. Figure 2B depicts the appearance of smooth and flexible ultra-thin sheets with slightly folded wrinkles on the porous carbon surface, indicating the reduction of GO to rGO. After deposition, uniformly and intensely dispersed AuNPs with an average diameter of ~50 nm can be clearly observed on the surface of rGO sheets, as shown in Figure 2C, demonstrating an improvement in the dispersion and high loading density of AuNPs. The SEM image of further electrochemically deposited MnO_2_ on the rGO/AuNPs/SPCE reveals a wall network-like structure and randomly dispersed larger nanoparticles (100~150 nm), sometimes appearing to be rods on the rGO sheet, as well as some particles in flower-like shapes, which is attributed to their combination with AuNPs (Figure 2D). The grain analysis of the SEM micrographs was further performed through statistical parameters, using the SEM image processing software Image-Pro Plus. The results show that the mean size of AuNPs ranging from 40 to 65 nm, and MnO_2_ ranging from 110 to 140 nm, are indicative of fine to medium size. A high-resolution SEM image of the electrodeposit-modified SPCE (enlarged partial view in the Figure 2D) also indicates that both AuNPs and MnO_2_ nanoparticles, as well as their flower-like composites, are well anchored onto flaky rGO on the porous carbon surface, which is similar to the morphologies observed in related Au/rGO or MnO_x_/rGO-modified electrodes [35,36]. In addition, Figure S2 portrays the energy dispersive X-ray spectrum (EDS) for rGO/AuNPs/MnO_2_/SPCE. The results reveal that the nanocomposite material deposited on the SPCE mainly consists of C, O, Au, and Mn elements, which further confirms the successful fabrication of rGO/AuNPs/MnO_2_/SPCE. Moreover, XPS was used to investigate the composition of the rGO/AuNP/MnO_2_ nanocomposites. As shown in Figure S3, the XPS full spectra showed peaks at 638.31, 527.38, 281.43, and 84.32 eV, which can be ascribed to Mn2p, O1s, C1s, and Au4f, respectively. Meanwhile, the percentage contents of Mn, O, C, and Au elements in the nanocomposite were measured to be 8.1%, 25.57%, 58.75%, and 0.83%, respectively. Both results of the XPS and EDX mapping are in complete agreement.

**Figure 3:**
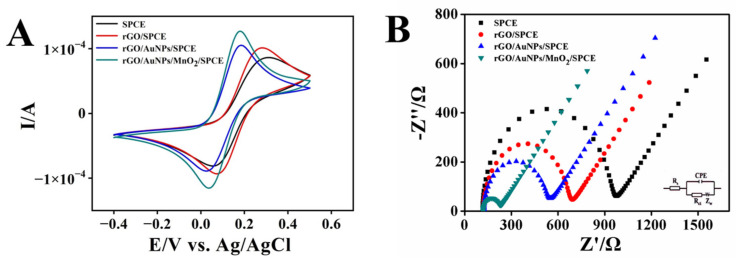
The CV curves (**A**) and Nyquist plots of EIS (**B**) of the bare SPCE, rGO//SPCE, rGO/AuNPs/SPCE, and rGO/AuNPs/MnO_2_/SPCE in 5 mmol L^−1^ [Fe (CN)_6_]^3−/4−^ containing 0.1 mol L^−1^ KCl.

CV and EIS were employed to characterize the electrochemical performance of the bare and nanocomposite-modified electrodes in a 5 mmol L^−1^ [Fe (CN)_6_]^3−/4−^ solution containing 0.1 mol L^−1^ KCl. As shown in Figure 3A, [Fe (CN)_6_]^3−/4−^ as the electrochemical marker, generated a pair of oxidation/reduction peaks at all electrodes with peak potential separation (ΔE_P_) larger than 120 mV and the peak current ratio (I_pa_/I_pc_) of about one, indicating a quasi-reversible oxidation/reduction process of the Fe^2+^/Fe^3+^ species. In addition, the peak potential differences in bare SPCE, rGO/SPCE, rGO/AuNPs/SPCE, and rGO/AuNPs/MnO_2_/SPCE gradually decreased from 227 to 136 mV, demonstrating that the deposition of rGO/AuNP/MnO_2_ nanocomposites on SPCE did facilitate a faster electron transfer than other nanomaterials toward the electrode in solution. Furthermore, it was found that the peak current of Fe^2+^/Fe^3+^ species obtained from rGO/SPCE, rGO/AuNPs/SPCE, and rGO/AuNPs/MnO_2_/SPCE increased successively, as compared to that of bare SPCE, which can be attributed to a larger active surface area offered by the modified nanomaterials on the SPCE. The results also show a drastic acceleration of the electron transfer rate between the nanocomposite electrode surface and the solution.

Figure S4 shows the relationship between the CV response of rGO/AuNPs/MnO_2_/SPCE and the scan rate. It can be observed that both the oxidation and reduction peak currents of [Fe (CN)_6_]^3−/4−^ obtained at the electrode are proportional to the square root of the scan rate, indicating that the electrode reaction was controlled by the diffusion process. According to the Randles–Sevčik equation (Equation (5)):

I_p_ = (2.69 × 10^5^) n^3/2^D^1/2^*v*^1/2^A*c* (5)

where I_p_ represents the peak current (A), n is the electron transfer number, D is the diffusion coefficient of the redox probe in the solution (cm^2^ s^−1^), *v* is the scan rate (V s^−1^), A stands for the electroactive surface area of the electrode (cm^2^), and *c* is the concentration of redox probe (mol L^−1^). The electroactive surface area of rGO/AuNPs/MnO_2_/SPCE calculated from this equation is 0.11 cm^2^, which is significantly larger than that of bare SPCE (0.06 cm^2^). These results indicate that the electrochemical performance of SPCE can be greatly enhanced by the electrodeposition of rGO/AuNPs/MnO_2_ nanocomposites.

The charge transfer resistance (R_cti_) of bare SPCE and different nanomaterial-modified electrodes were also measured by EIS, depending on their linearity with the semicircle diameter in the Nyquist diagrams (Figure 3B). The R_ct_ of bare SPCE, rGO/SPCE, rGO/AuNPs/SPCE, and rGO/AuNPs/MnO_2_/SPCE is found to be 823.6 Ω, 543.6 Ω, 397.8 Ω, and 99.54 Ω, respectively, showing the most remarkable synergistic effect of rGO, AuNPs, and MnO_2_ for promoting the electron transfer process.

### 3.2. Electrochemical Responses of rGO/AuNPs/MnO_2_/SPCE toward As(III)

**Figure 4:**
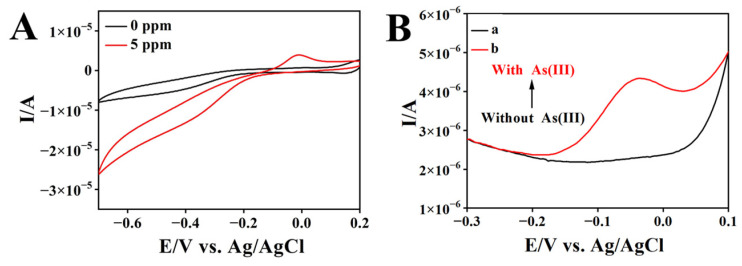
Cyclic voltammograms of rGO/AuNPs/MnO_2_/SPCE in 0.01 mol L^−1^ H_2_SO_4_ solution with different concentrations of As(III) (**A**); SWASV diagram of rGO/AuNPs/MnO_2_/SPCE in 0.01 mol L^−1^ H_2_SO_4_ solution without (curve a) and with (curve b) 100 ppb As(III) (**B**).

The electrochemical behavior of As(III) on rGO/AuNPs/MnO_2_/SPCE in 0.01 mol L^−1^ H_2_SO_4_ solution was examined using CV. As shown in Figure 4A, with the addition of As(III), an insignificant reduction peak was observed at −0.35 V (vs. Ag/AgCl), and a notable oxidation peak appeared at −0.05 V, which might be attributed to the electroreduction of As(III) to As(0) and the redissolution of As(0) to As(III) [37]. The obvious stripping response of As(0) at the nanocomposite electrode suggests the possible high-sensing sensitivity of As(III).

Owing to its high sensitivity over CV, SWASV was employed to examine the analytical performance of rGO/AuNPs/MnO_2_/SPCE toward As(III). Figure 4B shows the SWASV diagrams of rGO/AuNPs/MnO_2_/SPCE for blank electrolyte solution (curve a) and 100 ppb As(III) (curve b) in a 0.01 mol L^−1^ H_2_SO_4_ solution. It is obvious that a dissolution peak appeared at near −0.05 V with the addition of As(III), which is assigned to the electrooxidation of deposited As (0) to As(III) species in the stripping step.

To further evaluate the detection performance of rGO/AuNPs/MnO_2_/SPCE, SWASV responses of 100 ppb As(III) on different modified electrodes (SPCE, rGO/SPCE, rGO/AuNPs/SPCE, and rGO/AuNPs/MnO_2_/SPCE) were recorded and compared. As shown in Figure S5, the observed stripping current increased in order by the type of composite (I_SPCE_ < I_rGO/SPCE_ < I_rGO/AuNPs/SPCE_ < I_rGO/AuNPs/MnO2/SPCE_), demonstrating the excellent response of rGO/AuNPs/MnO_2_/SPCE electrodes in detecting As(III). The remarkable enhancement of the rGO/AuNPs/MnO_2_ nanocomposites toward As (III) may be attributed to the electrocatalytic properties of nanocomposites that derive from the synergetic effect of AuNPs, MnO_2_ NPs, and rGO. It can be noticed that the reduction of GO to rGO on SPCE not only enlarged the surface area of the electrode, but also provided residual oxygenous active groups that can form a complex with As(III) and enhance detection sensitivity [38]. Further, the electrodeposition of AuNPs also leads to the formation of Au–As intermetallic compounds on the surface of nanocomposite-modified SPCE, thus notably promoting the redox process of As(III) [39]. Although the conductivity of the deposited MnO_2_ layer is somewhat limited, its high adsorption capability and stability did contribute to the higher sensing performance of the nanocomposite electrode [40,41].

**Figure 5:**
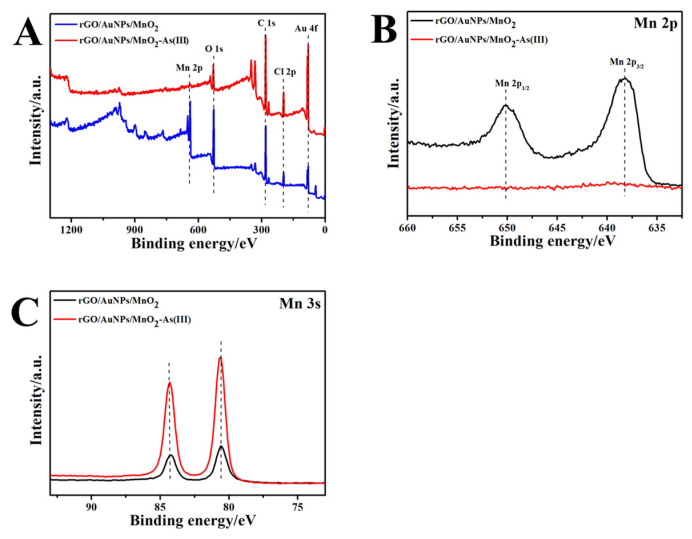
XPS spectra of rGO/AuNPs/MnO_2_ (**A**), Mn 2p (**B**), and Mn 3s (**C**) before and after electroreduction of As(III) on the rGO/AuNPs/MnO_2_/SPCE.

In addition, XPS was used to analyze the changes in the nanocomposite-modified electrode before and after the in situ electro-adsorption of As(III). As shown in Figure 5, the Mn2p peak almost completely disappeared after the electro-adsorption of As(III) at the rGO/AuNPs/MnO_2_/SPCE, while the Mn3s multiplet splitting peaks increased significantly, indicating a corresponding change in the valence state of the Mn element. These phenomena suggest that MnO_2_ is directly involved in the electrochemical reduction step of As(III) and also acts as the center of electrocatalytic reaction rather than simple physical adsorption. 

Therefore, the mechanism for electrochemical sensing of As(III) by rGO/AuNPs/MnO_2_/SPCE can be further speculated. Firstly, the integration of rGO as a sensitive layer matrix not only increases the surface area and conductivity of the electrode, but also significantly promotes the dispersion of AuNPs and MnO_2_. Secondly, the free As(III) in the solution can be enriched on the electrode surface either in the form of Au–As intermetallic compounds (via AuNPs) or through physical and chemical adsorption (via MnO_2_). Subsequently, the electrochemical reduction of As(III) to As(0), as well as the anodic stripping of As(0), can be largely accelerated by the involvement of MnO_2_ and AuNPs with excellent electrocatalytic properties on the nanocomposite electrode. In this case, the stripping peak current of As(III) will be significantly enhanced.

### 3.3. Optimization of Sensing Conditions toward As(III)

To obtain the maximum electrochemical response for As(III) detection with rGO/AuNPs/MnO_2_/SPCE, several experimental parameters were optimized in 0.01 mol L^−1^ H_2_SO_4_ using SWASV, including the cycles for electrodeposition of MnO_2_, the supporting electrolyte, deposition potential, and deposition time.

The influence of the cycles for electrodeposition of MnO_2_ on the stripping current of arsenic species was studied. As shown in Figure S6A, the peak current was enhanced when the number of deposition cycles increased from 1 to 2, indicating that the initial deposition of MnO_2_ NPs on rGO/AuNPs/SPCE increased the number of adsorption sites. However, a further increase in the number of deposition cycles resulted in an excessive deposition of MnO_2_ on the surface of the nanocomposite electrodes, which will affect the conductivity of the electrode and decrease the peak current. Therefore, two cycles were selected for the deposition of MnO_2_.

Figure S6B depicts the influence of H_2_SO_4_ concentration on the stripping peak current of As(III) detection. It is clear that the magnitude of the peak current is determined by the concentration of the supporting electrolyte, and the maximal response was achieved in 0.01 mol L^−1^ H_2_SO_4_, due to the fact that the reduction of H_3_AsO_3_ to As(0) can be promoted at an appropriate H^+^ concentration. Whereas too high a concentration of H_2_SO_4_ will lead to slower electron transfer kinetics, thereby reducing the peak current [42]. 

As shown in Figure S6C, the stripping current increased obviously when the deposition potential negative shifts from −1.2 to −1.4 V, which is attributed to the improved reduction of arsenite ions at a more negative potential. By a further negative shift in deposition potential, a sharp decrease in stripping current was found, suggesting the occurrence of hydrogen reduction, which will create bubbles and reduce the effective electrode surface area. In this case, an unstable response with larger background currents was observed. Therefore, −1.4 V was chosen as the deposition potential for further experiments.

Figure S6D demonstrates the influence of deposition time ranging from 60 to 240 s on the peak current of As(III), which shows a gradual increase in current signal at 180 s. When the deposition time exceeded 180 s, the current was observed to be decreased, which is assigned to the surface saturation of the nanocomposite electrode by the As(0). Meanwhile, an obvious decrease in electrode stability and reproducibility was observed at higher deposition times. Therefore, 180 s was selected as the best deposition time to avoid interference. It is clear that the fabrication of the proposed nanocomposite electrode required a shorter deposition time than its other competing materials based on AuNPs and rGO/Fe_3_O_4_ [38,43].

### 3.4. Analytical Performance of rGO/AuNPs/MnO_2_/SPCE for As(III) Detection

**Figure 6:**
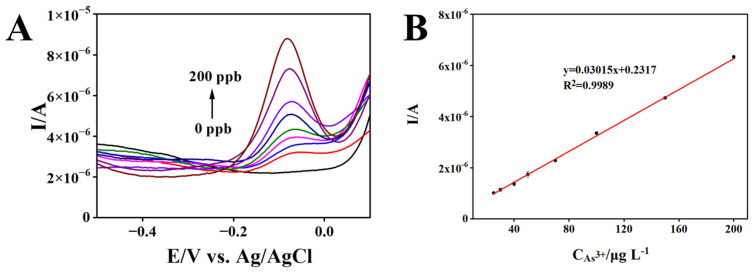
SWASV responses of rGO/AuNPs/MnO_2_/SPCE toward different concentrations of As(III) in 0.01 mol L^−1^ H_2_SO_4_ solution (**A**). The corresponding calibration plots for As(III) detection (**B**).

Under optimized SWASV sensing conditions, a calibration experiment was conducted, and the results are presented in Figure 6. As shown in Figure 6A, the stripping peak current of As(III) gradually increased with the increase in As(III) concentration. Figure 6B presents a good linear relationship between peak current and As(III) concentration in the range of 25–200 μg L^−1^. The detection limit is calculated to be 2.4 μg L^−1^ (RSD 4.7%), based on the signal-to-noise of three, and the sensitivity of the proposed sensor is 0.03015 μA·μg^−1^ L (RSD 4.7%). As listed in Table 1, the rGO/AuNPs/MnO_2_/SPCE electrode exhibits good durability and rapid preparation in comparison with the other reported modified electrodes, and a satisfactory detection limit that was well below the WHO guidelines for drinking water. Most notable is that the proposed sensor provides a fast response, stable, low-cost, and field-deployable platform for real-time monitoring of As(III) in water.

### 3.5. Selectivity, Reproducibility, and Stability Measurement

**Figure 7:**
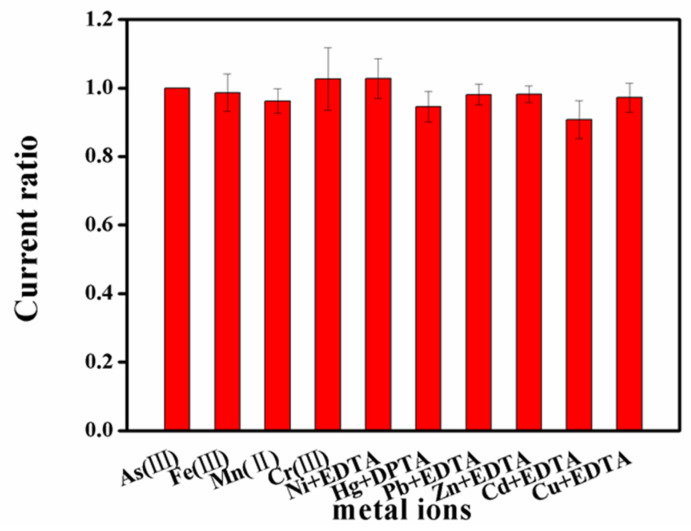
Ratio of stripping peak current of 100 ppb As(III) to interfering metal ions on rGO/AuNPs/MnO_2_/SPCE.

To evaluate the selectivity of the proposed rGO/AuNPs/MnO_2_/SPCE sensor, 100 ppb As(III) was detected by SWASV in 0.01 mol L^−1^ H_2_SO_4_ containing 10-foldother metal ions that might interfere with detection in the water sample, including Fe(III), Mn(II), Cr(III), Ni(II), Hg(II), Cd(II), Pb(II), Zn(II), and Cu(II). As can be seen from Figure 7, a not significant change in peak current was observed with the addition of Fe(III), Mn(II), and Cr(III). Although the presence of equivalent other interfering ions affected the peak current of As (III) due to peak overlapping or suppression; these interferences can be eliminated by adding a dose of EDTA or DTPA before water analysis.

Due to the inkjet printing preparation and modification of SPCE, it is necessary to evaluate the reproducibility (different batches) and repeatability (repetitively measuring on the same sensor) of the proposed SPCE-based sensors. As shown in Table S2, good repeatability and reproducibility (RSD < 5%) of the sensor were obtained at three As(III) concentration levels. Furthermore, the sensor can be regenerated with an electrochemical cleaning procedure by applying the corresponding potential. In addition, the sensor maintains the detection signal at 90% of its initial value after storage at 4 °C for 42 days, indicating excellent long-term stability. 

### 3.6. Sample Analysis

To evaluate the practical applicability of the proposed sensor, various drinking water samples were utilized for the SWASV measurement of As(III) under optimal conditions. The results showed that no As(III) was detected in blank water samples. Three water samples were then spiked with As(III) at three concentration levels and used for analysis in the recovery experiments. As listed in Table 2, the recoveries obtained from the spiked water samples ranged from 92.8% to 114.6%, demonstrating the accuracy and reliability of As(III) detection by this sensing method.

## 4. Conclusions

In summary, a facile in situ electrodeposition method was developed to modify rGO/AuNP/MnO_2_ nanocomposites onto SPCE for rapid As(III) sensing by SWASV in water samples. The nanocomposite electrode shows attractive sensing performance toward As(III) in terms of good sensitivity with LOD within the WHO guidelines, low-cost, and stability. In addition, as compared to the other arsenic sensor, the largely reduced pre-enrichment time (180 s), portability, and reinforced long-term stability (42 days) represent another figure of merit of the as-prepared sensor, which may favor in-field detection. The satisfied analytical capability can be attributed to a synergistic effect of the electrocatalytic properties of AuNPs and MnO_2_ NPs, strong adsorption capabilities, and the stability of MnO_2_, as well as the large surface area of the rGO-based composite. The proposed sensor was applied to the analysis of trace As(III) in real water samples with satisfactory recoveries. These findings offer a promising application potential for the field-deployable and cost-effective monitoring of As(III) in water.

## Figures and Tables

**Table 1:** Comparison of the analytical performances for As(III) detection using different electrochemical sensors.

Electrode	Technique	Modification Method	LDR (μg L^−1^)	LOD (μg L^−1^)	Service Life (days)	Reference
AuNPs/SPCE	ASV	Electrodeposition	30–150	8.9	-	[44]
Fe_3_O_4_/AuNPs/GCE	SWASV	Drop casting	1–100	0.22	-	[45]
PDDA/AuNPs/GCE	DPV	Layer-by-layer assembly	0–7492	4.36	-	[46]
MnFe_2_O_4_/AuNPs/GCE	SWASV	Drop casting	10–110	3.37	10	[47]
Fe_3_O_4_/rGO/GCE	SWV	Drop casting	1–20	1.19	15	[48]
Ag/SPCE	SWASV	Electrodeposition	10–80	8.4	-	[49]
AuNPs/CeO_2_-ZrO_2_/GCE	SWASV	Drop casting	0.5–15	0.137	-	[12]
Au_89_Cu_11_/GCE	SWASV	Drop casting	10–100	2.09	-	[50]
AuNPs/rGO/CPE	ASV	Electrodeposition	1–20	0.13	30	[28]
Fe_3_O_4_/Co_3_S_4_/SPCE	SWASV	Drop casting	1–10	0.691	-	[51]
AuNPs/CNTs/GCE	SWV	Drop casting	0.75–7.5	0.1	-	[18]
AuNPs/Co_3_O_4_/SPCE	SWASV	Drop casting	0.1–1/1–20	0.09/0.79	-	[52]
rGO/AuNPs/MnO_2_/SPCE	SWASV	Electrodeposition	25–200	2.4	42	This work

**Table 2:** Detection of As(III) in different water samples using the proposed sensor. (*n* = 3).

Sample	Added (μg L^−1^)	Found (μg L^−1^)	Recovery (%)	RSD (%)
Tap water	50.0	53.38	106.8	2.7
	100.0	110.3	110.3	7.6
	150.0	139.1	92.8	8.5
Mountain spring water	50.0	57.28	114.6	9.4
	100.0	108.3	108.3	9.2
	150.0	158.2	105.5	3.0
mineral water	50.0	48.72	97.4	7.9
	100.0	105.6	105.6	8.8
	150.0	163.8	109.2	4.7

## Data Availability

Not applicable.

## Supplementary Materials

The following supporting information can be downloaded at: https://www.mdpi.com/article/10.3390/bios13050563/s1, Figure S1. Cyclic voltammograms for (A) synthesis of rGO/SPCE in 1.0 mg mL^−1^ graphene oxide dispersion (0.1 mol L^−1^ pH 9.0 CBS). Segments: 8. Scan rate: 50 mV/s; (B) synthesis of rGO/AuNPs/SPCE via constant potential deposition in 1.0 mmol L^−1^ HAuCl_4_ (0.2 mol L^−1^ Na_2_SO_4_). Deposition potential: −0.2 V. Deposition time: 90 s; (C) synthesis of rGO/AuNPs/MnO_2_/SPCE in 0.05 mol L^−1^ C_4_H_6_MnO_4_•4H_2_O (0.1 mol L^−1^ Na_2_SO_4_). Segments: 2. Scan rate: 50 mV/s. Figure S2. EDS images of rGO/AuNPs/MnO_2_/SPCE. Figure S3. Cyclic voltammograms of 5 mmol/L Fe(CN)_6_^3−/4−^ in a 0.1 mol/L KCl solution recorded on rGO/AuNPs/MnO_2_/SPCE at different scan rates (A); Corresponding linear relationship between the redox peak currents and the scan rate (B). Figure S4. SWASV responses for 100 ppb As(III) in 0.01 mol L^−1^ H_2_SO_4_ solution on the bare SPCE, rGO/SPCE, rGO/AuNPs/SPCE, and rGO/AuNPs/MnO_2_/SPCE. Deposition potential: −1.4 V; deposition time: 180 s. Figure S5. The influences of deposition cycles for MnO_2_(A), pH(B), deposition potential(C), and deposition time(D) on the stripping peak current of 100 ppb As(III). Table S1. The reproducibility of as-prepared SPCE-based sensor for the detection of As(III) (*n* = 3).
