# Supplementary material for: Electrodeposited rGO/AuNP/MnO2 Nanocomposite-Modified Screen-Printed Carbon Electrode for Sensitive Electrochemical Sensing of Arsenic(III) in Water
Source: Biosensors (Basel). 2023 May 21;13(5):563. doi: 10.3390/bios13050563 (PMC10216768; doi:10.3390/bios13050563)
Supplement: Supplementary file 1 [file biosensors-13-00563-s001.zip › biosensors-2370354-supplementary.pdf]

biosensors MDPI

*Supplementary Material*

# Electrodeposited rGO/AuNP/$MnO_2$ Nanocomposite-modified Screen-Printed Carbon Electrode for Sensitive Electrochemical Sensing of Arsenic(III) in Water

**Yanqing Wu, Tao Zhang, Lishen Su and Xiaoping Wu ***

Key Laboratory for Analytical Science of Food Safety and Biology (Ministry of Education & Fujian Province), College of Chemistry, Fuzhou University, Fuzhou 350116, China; lalalawuuu@163.com (Y.W.); taozhang202304@163.com (T.Z.); 201310017@fzu.edu.cn (L.S.)
* Correspondence: wapple@fzu.edu.cn

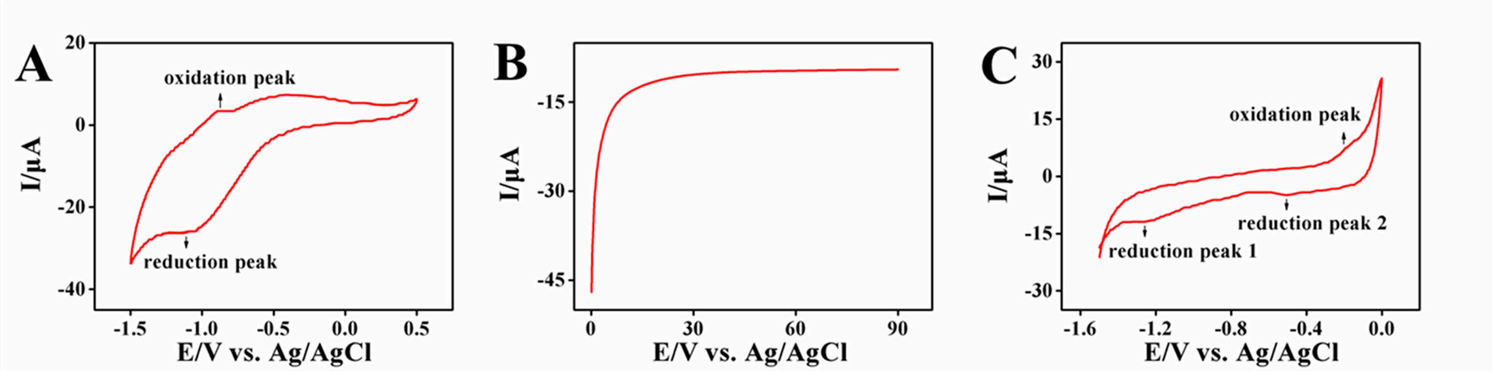


**Figure S1.** Cyclic voltammograms for (A) synthesis of rGO/SPCE in 1.0 mg $mL^{-1}$ graphene oxide dispersion (0.1 mol $L^{-1}$ pH 9.0 CBS). Segments: 8. Scan rate: 50 mV/s; (B) synthesis of rGO/AuNPs/SPCE via constant potential deposition in 1.0 mmol $L^{-1}$ $HAuCl_4$ (0.2 mol $L^{-1}$ $Na_2SO_4$). Deposition potential: –0.2 V. Deposition time: 90 s; (C) synthesis of rGO/AuNPs/$MnO_2$/SPCE in 0.05 mol $L^{-1}$ $C_4H_6MnO_4{\bullet}4H_2O$ (0.1 mol $L^{-1}$ $Na_2SO_4$). Segments: 2. Scan rate: 50 mV/s.

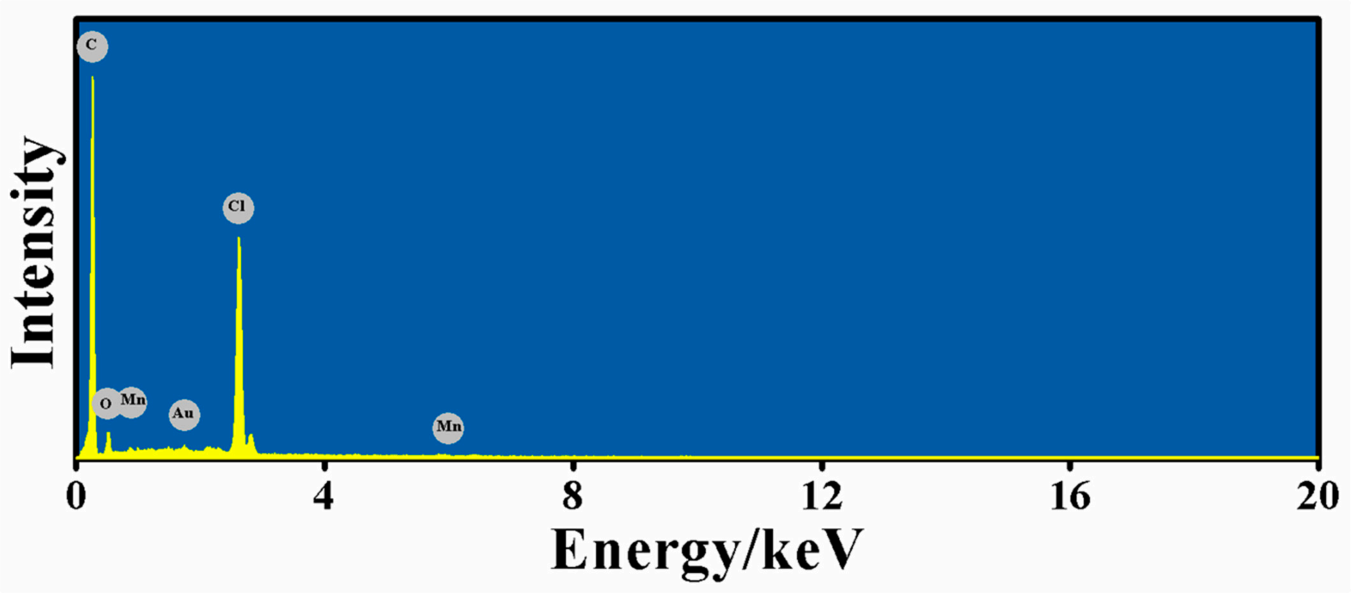


**Figure S2.** EDS images of rGO/AuNPs/$MnO_2$/SPCE.

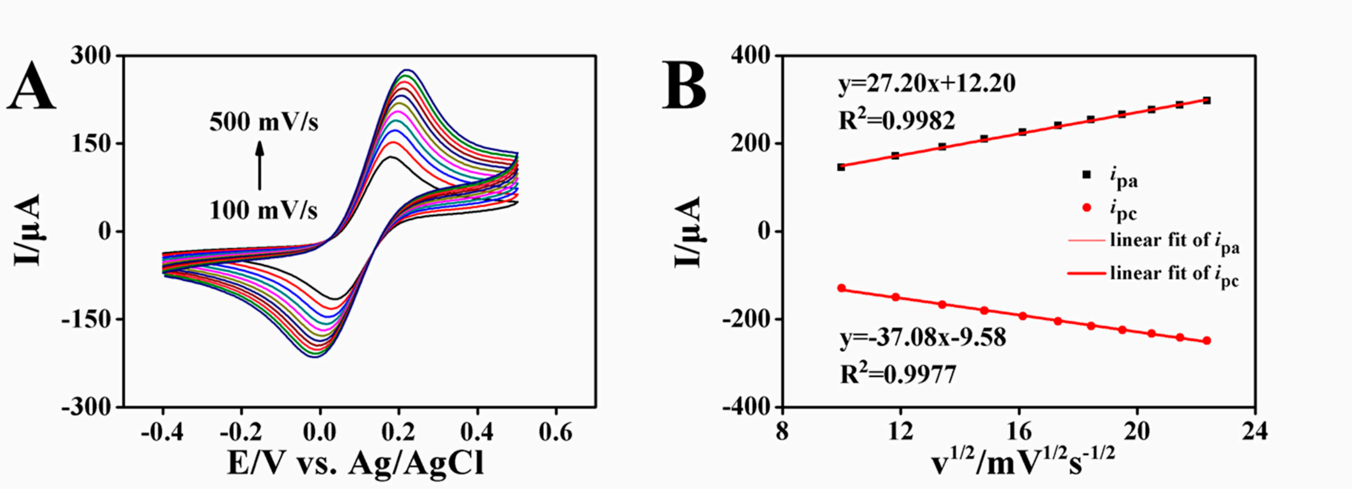


**Figure S3.** Cyclic voltammograms of 5 mmol/L $Fe(CN)_6^{3-/4-}$ in a 0.1 mol/L KCl solution recorded on rGO/AuNPs/$MnO_2$/SPCE at different scan rates (A); Corresponding linear relationship between the redox peak currents and the scan rate (B).

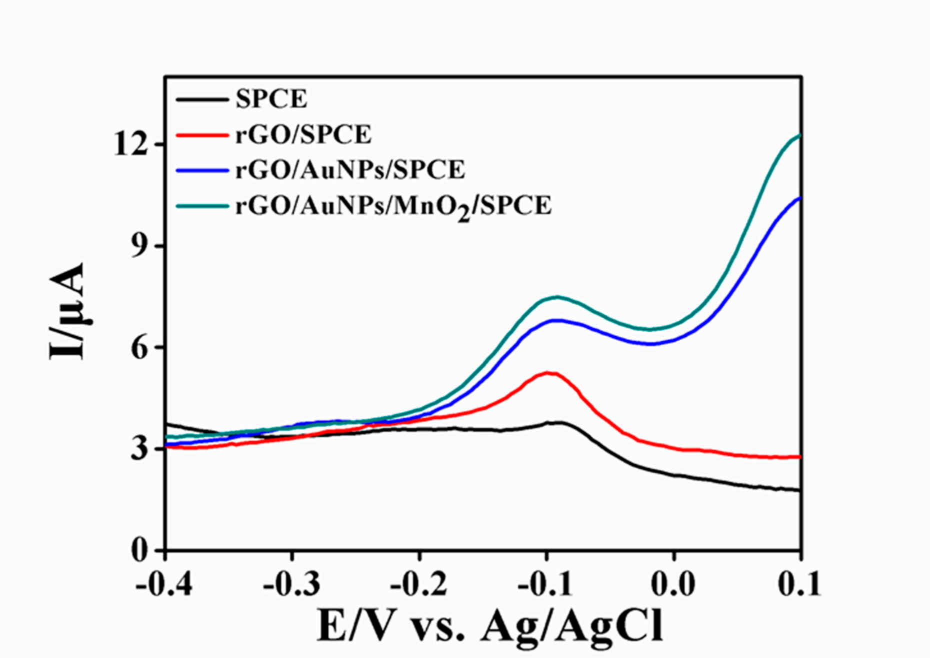


**Figure S4.** SWASV responses for 100 ppb As(III) in 0.01 mol $L^{-1}$ $H_2SO_4$ solution on the bare SPCE, rGO/SPCE, rGO/AuNPs/SPCE and rGO/AuNPs/$MnO_2$/SPCE. Deposition potential: –1.4 V; deposition time: 180 s.

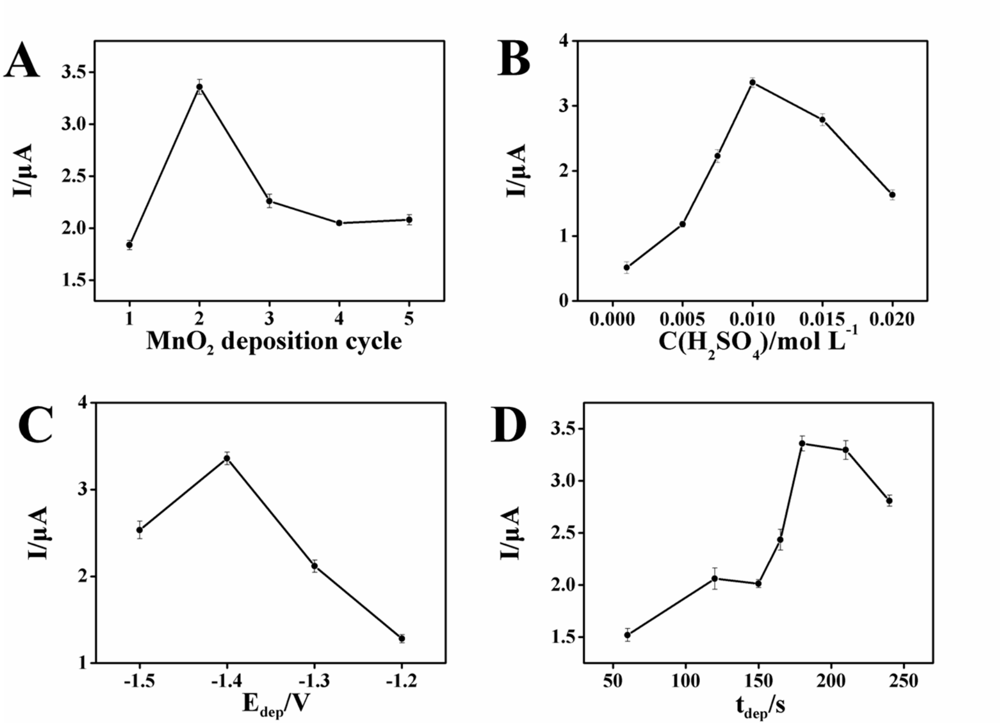


**Figure S5.** The influences of deposition cycles for MnO2(A), pH(B), deposition potential(C) and deposition time(D) on the stripping peak current of 100 ppb As(III).

**Table S1.** The reproducibility of as-prepared SPCE-based sensor for the detection of As(III)(n=3).

| Sample number | Concentration of As(III) (μg $L^{-1}$) | Run-to-run RSD (%) | Batch-to-batch RSD (%) |
|---|---|---|---|
| 1 | 50 | 3.5 | 4.9 |
| 2 | 100 | 4.1 | 4.4 |
| 3 | 150 | 5.0 | 4.3 |
